# Supplementary figures and images for: A varying T cell subtype explains apparent tobacco smoking induced single CpG hypomethylation in whole blood
Source: Clin Epigenetics. 2015 Aug 6;7(1):81. doi: 10.1186/s13148-015-0113-1 (PMC4526203; doi:10.1186/s13148-015-0113-1)

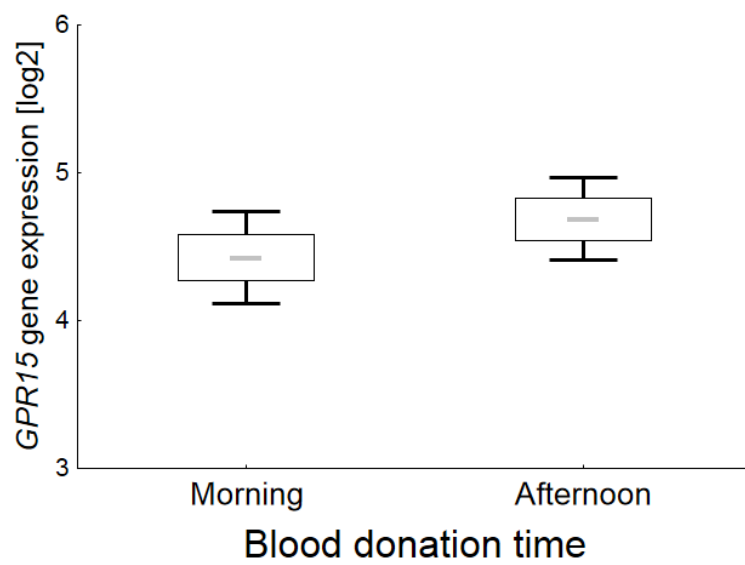

Supplement: Additional file 2: — A figure showing “GPR15 gene expression versus circadian rhythm. For the Working Place Cohort, peripheral blood was collected before (morning) and after (afternoon) work”. [file 13148_2015_113_MOESM2_ESM.pdf]

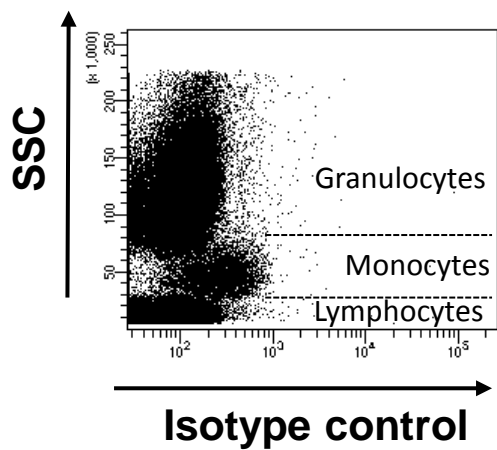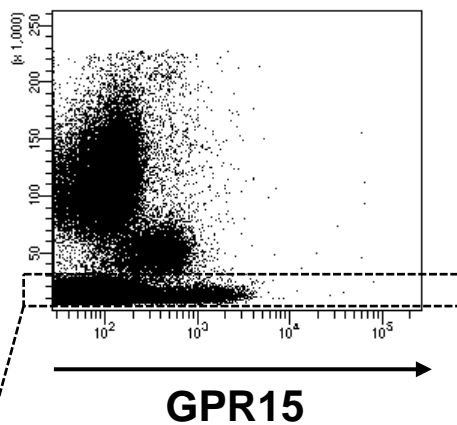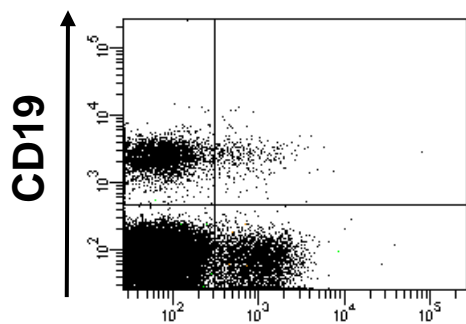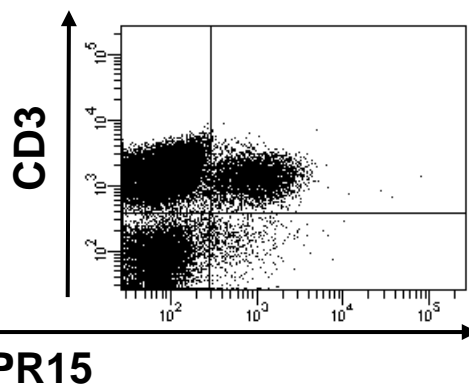

Supplement: Additional file 3: — A figure showing “Representative dot plots for the gating strategy for isotype control and GPR15 staining. Gated lymphocytes were further characterized for the expression of CD3 and CD19. [file 13148_2015_113_MOESM3_ESM.pdf]
